# Plate Food Waste in Food Services: A Systematic Review and Meta-Analysis

**DOI:** 10.3390/nu16101429

**Published:** 2024-05-09

**Authors:** Nathalia Sernizon Guimarães, Marcela Gomes Reis, Luciano de Alvarenga Fontes, Renata Puppin Zandonadi, Raquel Braz Assunção Botelho, Hmidan A. Alturki, Ariana Saraiva, António Raposo

**Affiliations:** 1 Department of Nutrition, Nursing School, Universidade Federal de Minas Gerais, Alfredo Balena Avenue, 190, Room 314, Santa Efigênia, Belo Horizonte 30130-100, Minas Gerais, Brazil; reis.marcelanutri@gmail.com; 2 Department of Agricultural Engineering, Vaz de Mello Consultoria e Perícia. Gonçalves Dias Street, 1181, Funcionários, Belo Horizonte 30140-091, Minas Gerais, Brazil; consultorfundiario@gmail.com; 3 Department of Nutrition, School of Health Sciences, University of Brasilia (UnB), Campus Darcy Ribeiro, Asa Norte 70910-900, Brasilia, Brazil; renatapz@unb.br (R.P.Z.); raquelbotelho@unb.br (R.B.A.B.); 4 King Abdulaziz City for Science & Technology, Wellness and Preventive Medicine Institute—Health Sector, Riyadh 11442, Saudi Arabia; halturki@kacst.edu.sa; 5 Department of Animal Pathology and Production, Bromatology and Food Technology, Faculty of Veterinary, Universidad de Las Palmas de Gran Canaria, Trasmontaña s/n, 35413 Arucas, Spain; ariana_23@outlook.pt; 6 CBIOS (Research Center for Biosciences and Health Technologies), Universidade Lusófona de Humanidades e Tecnologias, Campo Grande 376, 1749-024 Lisboa, Portugal

**Keywords:** food waste, food services, sustainability, collective feeding

## Abstract

Food waste is considered to be a social, environmental, administrative, and economic problem. Given the large-scale production and distribution of food, food waste in food services has been widely discussed by experts, professors, and scientists in the field. This systematic review aimed to understand which food service has the highest percentage of plate food waste. A systematic review and meta-analysis were conducted until January 2024 in ten electronic databases: MEDLINE, Embase, IBECS, BINACIS, BDENF, CUMED, BDNPAR, ARGMSAL, Cochrane Library, Sustainable Development Goals, and the gray literature. The protocol was previously registered with PROSPERO under the code CRD42024501971. Studies that have assessed plate food waste in food services were included. There were no restrictions on language, publication location, or date. The risk of bias analysis was carried out using the JBI instrument. A proportion meta-analysis was carried out using R software (version 4.2.1). This systematic review with meta-analysis showed that the type of distribution and the food service are the factors that have the greatest impact on the percentage and per capita of plate food waste. In the face of increased waste, interventions should be targeted by type and distribution system, diners, and meals in order to lessen the impact of these factors.

## 1. Introduction

Food services include commercial and institutional establishments, and they aim to manage the production of nutritionally balanced meals with good hygienic and sanitary standards for consumption outside the home. They may contribute to maintaining or recovering the health of groups and help to develop eating habits [1,2].

The success of a food service operation lies in the precise definition of its objectives, its administrative structure, its physical facilities and human resources, and, above all, the standardization of all the operations carried out, which must be supported by the five elements of the administrative process: forecasting, organization, command, coordination and control. Processes are a set of inter-related activities designed to optimize quality customer service. For a process to take place, the transformation of food and drink (input) into products/meals (outputs) must occur [3]. Given the production process carried out on a large scale in food services, the waste of food, water, materials, and energy, among other things, has been one of the biggest problems due to leftovers and food scraps [4].

In the area of food, the impact of waste is a social, environmental, administrative, and economic problem, leading to an annual global cost of USD 2.65 billion, so that almost a third of all food produced is wasted annually [5]. This not only represents a huge waste of natural resources such as water, energy, and land, but also contributes significantly to greenhouse gas emissions associated with food production. Studies show the relationship between waste and the reallocation of wasted food to cover hunger in various nations [6,7,8]. According to the data described by some studies, 10 tons of food that have been wasted could feed 12,470 people [9,10,11].

In this way, reducing food waste worldwide is directly associated with the amount of wasted food that could feed countless families in situations of hunger and food and nutritional insecurity. At a global level, food and nutritional insecurity affect not only low- and middle-income countries but also high-income countries such as the United States of America [12,13].

To quantify food waste, the percentage of leftovers, i.e., the ratio between the leftovers returned on the trays by the diner and the amount of food and food preparations offered, is used and expressed as a percentage. The control of leftovers aims to assess the adequacy of the quantities prepared concerning consumption needs, portioning in distribution, and acceptance of the menu. In healthy groups, less than 10% rates are acceptable as a percentage of leftover intake [14]. Food waste in food services can serve as a measure of the quality of the service. The variables of food seasonality and handler training should be considered in any food service that aims to optimize its actions in the use of food [15].

Considering that leftover food interferes in many social, environmental, and economic areas, resulting in significant impacts on sustainability, this systematic review aimed to understand which food service has the highest percentage of plate food waste. The data from this study will be important for adopting specific campaigns and actions according to the frequency of waste.

## 2. Materials and Methods

A systematic review and meta-analysis were carried out according to the recommendations of the Cochrane Collaboration [16] and written according to the PRISMA checklist [17]. The study protocol was previously registered on the PROSPERO platform under the code CRD42024501971.

### 2.1. Search Strategy

To answer the question “Does the frequency of food waste differ by type of food service?”, we searched ten different independent databases: MEDLINE (PubMed), Embase; Cochrane Library Collaboration; *Índice Bibliográfico Espanhol em Ciências de la Salud (IBECS), Bibliografía Nacional en Ciencias de la Salud Argentina (BINACIS), Base de dados de Enfermagem (BDENF), Committee on Undergraduate Medical Education (CUMED), Base de Datos Nacional del Paraguay (BDNPAR), Revista Argentina de Salud Pública (ARGMSAL),* and Sustainable Development Goals (SDGs). In addition, a manual search was carried out in the included reference lists to understand local studies published in journals not indexed in the databases evaluated.

There were no language, date, document type, or publication status restrictions to including records. The search for studies was carried out in January 2024 and included studies up to this date. The descriptors were identified in Medical Subject Headings (MeSHs), Health Sciences Descriptors (DeCSs), and Embase Subject Headings (Emtree). Subsequently, the descriptors were combined with the Boolean operator AND, while their synonyms were combined with the Boolean operator OR. The search strategy adopted for each database is presented in Table S1.

### 2.2. Outcomes

The primary outcomes were plate food waste (or leftover food intake) (%) and per capita plate food waste (or per capita leftover food intake) (kg), following Equations (1) and (2) [10]:

(1) $$\%\;plate\;food\;waste=\frac{weight\;of\;plate\;food\;waste\times 100}{weight\;of\;meal\;distributed}$$

(2) $$Per\;plate\;food\;waste\left(kg\right)=\frac{weight\;of\;plate\;food\;waste(kg)}{number\;of\;served\;meals}$$

### 2.3. Eligibility Criteria

Observational studies (cross-sectional, case-control, or cohort) and intervention studies were included. Studies at food services such as hospital food service, school canteens, restaurants, university restaurants, and popular restaurants that evaluated plate food waste were included. Experimental studies, case series or case reports, trials, reviews, in vitro or experimental animal studies, cost-effectiveness analyses, letters, comments, or editorials were excluded.

### 2.4. Study Selection and Data Extraction

The studies found in the electronic search of the databases were exported in “ris” format to the Rayyan Qatar Computing Research Institute application for systematic reviews [18]. Two reviewers (NSG, MGR) screened the studies independently to determine whether they met the inclusion criteria.

Two reviewers (NSG, MGR) independently examined the titles and abstracts to determine whether they met the inclusion criteria. After this stage, a textual analysis of the studies was carried out independently. An independent reviewer analyzed any discrepancies. To create the extraction table, the following data were collected: reference (author, year, title), study location, research design, follow-up period (weeks), population characteristics (type of food service, diners, distribution method, and system), type of menu served, number of served meals, definition plate food waste, and main results for the outcomes assessed.

### 2.5. Quality Assessment

The Joanna Briggs Institute (JBI) tool was used to assess the methodological quality of the systematic prevalence review [19]. Two researchers independently assessed the risk of bias in the chosen studies. Disagreements between reviewers regarding potential bias in specific studies were resolved through discussion, occasionally involving a third review author. Studies were classified as having a low risk of bias if the total score was up to 49.0%, moderate risk of bias if the score fell between 50.0% and 70.0%, and high risk of bias if it was above 70.0%. The risk of bias in each study is described in Table S2 [20].

### 2.6. Meta-Analysis

This meta-analysis estimated the proportion of food waste using the crude proportions (PRAW) method with random effect. We chose this method because it corrected for overestimations of the weight of studies with estimates very close to 0% or 100% [21]. Subgroup analyses were carried out by type of food service, diners, distribution method, food service management, type of meal, and distribution system. The random effects model assessed heterogeneity, the chi-squared test was applied with a significance of *p* < 0.10, and its magnitude was determined by the I-squared (I^2^). In the all analyses, a *p*-value < 0.05 was considered statistically significant. The analyses were carried out using the ‘Meta’ packages in the Rstudio software, version 4.2.1 (R: A Language and Environment for Statistical Computing, Vienna, Austria).

## 3. Results

**Figure 1:**
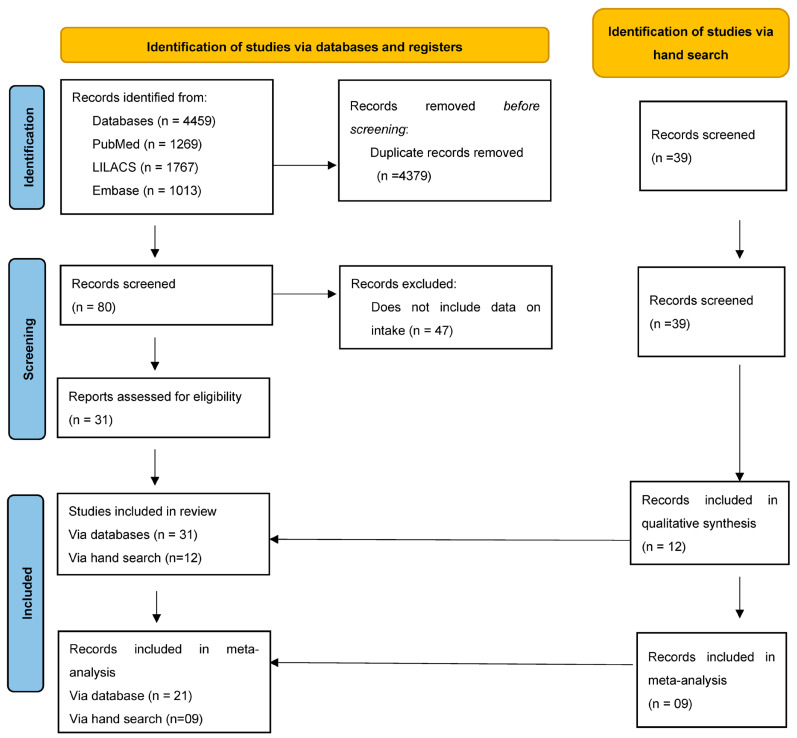
Flowchart for the selection of studies, 2024.

A total of 4459 studies were found. After excluding 4379 duplicates, 80 titles and abstracts were examined. Of these 80 records evaluated by full text, 49 were excluded according to the eligibility criteria, as described in Table S3. Further, 31 were included in the review studies via electronic database and 12 studies were added after a manual search of the gray literature. For the meta-analysis, in total of 21 studies via the electronic database and 9 of the gray literature were included. Therefore, 43 studies were included in the systematic review, and 30 studies were eligible for meta-analysis (Figure 1).

### 3.1. Characteristics of the Studies

Table 1 summarizes the main characteristics of the included studies. According to the location of the study, 32 (74.4%) were carried out in America (Brazil and USA), five (11.6%) in Asia (Indonesia, Taiwan, Libano, and China), five (11.6%) in Europe (Croatia, Denmark, Latvia, Lithuania, and Finland), and one in South Africa (2.32%).

### 3.2. Meta-Analysis

In the analysis of plate food waste (%), 30 studies were included, evaluating 117,819 meals and per capita plate food waste (kg). In the final column of Table 2, we have included the percentage of interpretation of the most frequent case within the subgroup evaluated in the meta-analysis to make it clearer. Studies not included in the meta-analysis due to lack of data are described in Table S3.

### 3.3. Plate Food Waste (in Percentual)

Table 2 summarizes the analysis of the included studies. Analyzing the percentage of plate food waste according to the type of food service, hospital food service (n = eight studies) is the type of service with the highest rate of plate waste (77.7%) and popular restaurants has the lowest rate (1.7%). The second highest percentage of plate food waste was observed in school canteens (n = five; 10.5%), commercial food service (n = ten studies; 7.3%), and university canteens (n = three studies; 2.7%), respectively.

Concerning the diners’ groups, diners and food service workers (n = five studies) had the highest percentage of plate waste, 69.95%, followed by diners and patient companions, 11.65%.

According to the forms of distribution, the study that only analyzed the distribution of meals in lunchboxes (n = 1 study) obtained a percentage approximately 3× higher, with 72.82% of plate waste, compared to the second highest type of waste, which would be distribution by plates, trays, and lunchboxes (n = 1 study) with 19.94%. With even lower percentages are distribution on plates (n = 15 studies; 3.33%), followed by trays (n = 4 studies; 1.85%), and the lowest percentage represented by the trays and plates subgroup (n = 4 study; 2.13%).

According to the type of management, they were classified as self-management (n = two studies) and outsourced (n = six studies). Self-management had the highest percentage of leftover food, with 87.40%, followed by outsourced management (12.60%).

According to the meal type, we used data from lunch (n = 19 studies), large meals (n = 6 studies), small and large meals and supper (n = 1 study), and snacks in general (n = 1 study). Supper accounted for the highest percentage of leftovers (77.87%), followed by small and large meals (7.56%), and lunch had the lowest percentage of plate waste food (2.91%).

According to the distribution system, they were classified as self-service (n = 8 studies), a la carte (n = 5 studies), and mixed (n = 12 studies), with the former having a higher percentage of plate waste (61.42%) and the latter a lower percentage of plate waste (16.42%).

### 3.4. Per Capita Waste (kg)

When analyzing the per capita number of leftovers according to the type of food service, it was possible to see that hospital food service and university canteens are the types of service with the highest per capita waste of leftovers (0.03 kg/per capita/meal). However, the other services, such as popular restaurants, school canteen food service, and commercial food service, obtained a per capita equal to zero, given the lower waste in their analysis (Table 3). The study by Chang and collaborators (2021) evaluating buffet restaurants [27] did not present per capita value.

Concerning the diners’ groups, the food service workers group obtained a per capita leftovers (per capita waste) of 0.01 kg, and the other subgroups, such as customers and employees as well as only customers, obtained a per capita equal to zero, given the lower waste in their analysis (Table 3).

According to the forms of distribution, the distribution in lunch boxes had a per capita leftover intake of 0.03 kg, followed by the trays and plates subgroup (0.02 kg), and 0.01 kg of the plates subgroup, and the distribution on plates, trays, and lunch boxes. It is worth noting that distribution on trays, due to their lower waste, accounted for zero kg in the analysis (Table 3). According to the management method, only the self-management had a per capita leftover different from zero (0.03 kg). For the type of meal, only lunch and large meals had a per capita different from zero (0.01 kg), while the snacks had zero kg/per capita. Even so, regarding the distribution system, both self-service and mixed service had 0.01 kg/per capita.

## 4. Discussion

Food waste is not only ethically unacceptable but has essential impacts on human health, food safety, and the environment. Plate food waste can be avoided, and its prevention is fundamental, but it depends on an individual’s awareness [61]. Studying data about plate food waste is essential to raise public awareness about the need for change.

This systematic review aimed to understand which food service has the highest plate food waste. It is estimated that in developing countries, food loss occurs mainly during the first stages of the food supply chain (post-harvest production and distribution due to lack of financial, technical, and management resources), while food waste in consumption tends to be lower than that of developed countries [61]. Despite not being studied, it probably occurs due to the food insecurity experienced in some developing countries and the concern about food waste in this context. Therefore, it is expected that there will be more studies on food waste in countries that suffer from food insecurity, as seen in this systematic review, in which around 70% of the studies were carried out in Brazil (Table 1).

This systematic review showed that hospital food service (n = nine studies) is the type of service with the highest rate of plate food waste (4.9%), and popular restaurants presented the lowest rate (0.07%). Hospital food service also has the highest per capita plate food waste (0.03 kg), which is justified by patients’ health conditions, the menus served, service, and hospital environmental issues [42]. These results can also be expected since hospital consumers are generally affected by illness or taking medications that can impair their appetite [28,41,45,48,49,52,57]. It is important to mention that five studies only evaluated the lunch meal, and others evaluated lunch and dinner or supper or all the meals. On the other hand, popular restaurants (or community restaurants) are part of a Brazilian assistance program that offers cheap and healthy meals to low-income populations. They mainly attend to people at risk of food insecurity, who are expected to eat all the food on their plates [62]. One study in this review was conducted in popular restaurants and evaluated just lunch. Therefore, it is difficult to compare studies because they served different types of meals, and the attending population is not the only criterion to be analyzed. The second highest percentage of plate food waste was observed in the school canteen food service (0.43%), which was also expected to be high since, in childhood, there is frequent food neophobia and a lack of sustainable and health knowledge, which can determine food choices, impact the quality of a diet, and influence unfinished plates [63]. Also, children are exposed to a greater variety of food in school canteens as part of the nutrition education process. Exposure to new ingredients and preparations is expected to cause more plate food waste.

Meal distribution in lunchboxes presented the highest percentage of plate food waste compared to distribution by plates and/or trays. Considering that lunchboxes are pre-prepared and do not allow the client to choose the dishes (and quantity) composing their meal, lunchbox food waste is expected to be higher than the distribution system in which clients may select dishes among served options and the amount that will compose their plate. Three studies evaluated lunchboxes in commercial restaurants, university restaurants, and school canteens.

Self-management food services presented a higher percentage of plate food waste (3.47%) than outsourced management (0.50%). Outsourced restaurants, that do not have their own management, need to comply with the criteria established by the contract manager, which are often associated with the menu’s quality aspects (nutritional, sensorial, microbiological, and economic). Furthermore, for a restaurant to make a profit, it needs to reduce waste in general, which involves good acceptance of the dishes by consumers. These aspects may explain the data from studies comparing outsourced and self-management restaurants.

According to the type of meal, supper accounted for the highest percentage of leftovers (5.35%). However, it is important to consider that the only study evaluating supper [42] and the study evaluating small and large meals [55] were performed in hospital restaurants, in which consumers are generally affected by illness or taking medications impairing their appetite in addition to being in the hospital environment [42]. Lunch presented the lowest percentage of plate waste food (0.27%). It is essential to highlight that only 26% (n = 5) of the studies only evaluated lunch and were performed in hospitals [28,45,49,52,57]. Almost half of the studies evaluating lunch were performed in restaurants in Brazil [11,23,31,38,42,46,47,51,59]. In Brazil, lunch is considered the main and largest meal during the day. It mainly comprises traditional and well-accepted dishes such as rice, beans, meat, and some vegetables. Considering the importance of lunch in Brazil and the food insecurity experienced in this country, these factors probably impacted the small percentage of lunch plate waste in this review.

Self-service restaurants had a higher percentage of plate food waste (0.86%), and mixed-service restaurants had a lower percentage (0.23%). A study showed that buffet (self-service) restaurants cause more plate food waste than other food services [64], which is similar to the findings in our review. Self-service restaurants can charge by plate weight or charge per person (regardless of the amount they will eat). When a meal is charged per person there tends to be greater waste, as the value is the same no matter how much food is put on the plate. When charged according to plate weight, consumers tend to be more attentive when choosing food and put less food on the plate, tending to create less food waste. However, many studies do not specify the type of self-service analyzed, which impairs discussion of this topic. However, the type of distribution service is a critical topic in plate food waste prevention, since this review showed it has the second greatest impact on the percentage of plate food waste.

It is important to highlight that most of the studies included in this review are from Brazil, which might skew the general applicability of the results to other global contexts, especially in countries with different eating habits and food service operations. It is also essential to note that the studies used different methodologies, which may affect the overall analysis, so the data must be analyzed cautiously. However, the knowledge about the type of food service, meal distribution system, and dinners that produce the most plate food waste may help managers plan educational actions to prevent and correct waste, as well as to identify the dishes that are most wasted (whether due to low acceptance, excessive portion size, or for another reason), allowing them to make changes to the menu to reduce plate food waste.

## 5. Conclusions

Plate food waste causes high financial waste, lower valuable nutrient intake by consumers, and a negative environmental impact. Several individuals’ factors may influence plate food waste, such as age, serving size, sex, food preferences, eating behaviors, competitive foods during meals, how long meals last, and educational and economic levels, among others. However, this review showed that aspects of food service also impact plate food waste. The type of distribution and the food service are factors that have the greatest impact on percentage and per capita of plate food waste. In contrast, the type and system of distribution, the types of diners, and the types of meals have less impact, but they are still relevant factors that need to be analyzed. Therefore, this review highlights the need for targeted interventions that reduce plate food waste and for understanding of the specific conditions of each food service type to help design effective waste reduction strategies.

## Figures and Tables

**Table 1:** Characteristics of the included studies.

Author and Year	Local	Design	Foodservice Type	Diners	Self-Managed or Outsourcing	Utensils	Distribution System	Meal	Period of Data Collection (Weeks)
Aranha et al., 2018 [9]	Brazil	Cross-sectional	n.i.	n.i.	n.i.	Trays	Mixed	Lunch	1
Augustini et al., 2008 [10]	Brazil	Cross-sectional	Restaurant	Food Service Workers	n.i.	Plate + Trays	Self-Service	Lunch + dinner	14
Barbosa et al., 2021 [22] *	Brazil	Cross-sectional	Restaurant	Food Service Workers	n.i.	Plate	Self-Service	n.i.	n.i.
Bardini et al., 2014 [23]	Brazil	Cross-sectional	n.i.	Food Service Workers	n.i.	Plate	Self-Service	Lunch	½
Bicalho et al., 2013 [24]	Brazil	Cohort	University Restaurant	n.i.	n.i.	Plate	Self-Service	Lunch	17
Borges et al., 2019 [25]	Brazil	Case report	University Restaurant	Diners + Food Service Workers	Outsourcing	Plate	Mixed	Lunch + dinner	17
Byker et al., 2014 [26]	USA	Cross-sectional	Primary School	Diners	n.i.	Plate	Self-Service	Lunch	1
Carvalho et al., 2013 [11]	Brazil	Cross-sectional	Restaurant	Food Service Workers	Outsourcing	n.i.	Self-Service	Lunch	1
Chang, 2021 [27]	Taiwan	Case-control	Restaurant	Diners	n.i.	Plate	Mixed	n.i.	
Chaves et al., 2019 [28]	Brazil	Cohort	Hospital Food Service	Food Service Workers	n.i.	Plate	v	Lunch	3
Coimbra et al., 2019 [29]	Brazil	Cross-sectional	University Restaurant	n.i.	n.i.	Plate	Mixed	Lunch	1
Dagiliūtė and Musteikytė, 2019 [30]	Lithuania	Cohort	Restaurant	Diners	Self-managed and outsourcing	Plate	Mixed	n.i.	24
Delazeri et al., 2015 [31] *	Brazil	Cohort	Restaurant	n.i.	n.i.	Trays	Self-Service	Lunch	1
Galego et al., 2014 [32] *	Brazil	Cross-sectional	n.i.	Food Service Workers	Self-managed	n.i.	n.i.	Lunch	2
Ilic et al., 2022 [33]	Croatia	Cross-sectional	Primary School	Diners	n.i.	Plate + Trays	A la carte	Lunch	1
Liu et al., 2016 [34]	China	Pilot study	Primary School	Diners	n.i.	Plate + Trays	A la carte	Lunch	n.i.
Lonska et al., 2022 [35]	Latvia	Cross-sectional	Primary School	Diners	n.i.	Plate + Trays	A la carte	Lunch	1
Machado et al., 2014 [36] *	Brazil	Case report	Restaurant	Food Service Workers	n.i.	Plate	n.i.	Lunch	2
Marais et al., 2017 [37]	South Africa	Cross-sectional	Restaurant	Diners + Food Service Workers	Outsourcing	n.i.	n.i.	Lunch + dinner	½
Matzembacher et al., 2020 [38] *	Brazil	Cohort	Restaurant	n.i.	Self-managed	Plate	Mixed	Lunch	4
Medeiros et al., 2014 [39]	Brazil	Cross-sectional	n.i.	n.i.	n.i.	Plate	n.i.	Lunch	½
Mello et al., 2011 [40]	Brazil	Cross-sectional	Restaurant	n.i.	Outsourcing	Plate	Mixed	Lunch + dinner	3
Nonino Borges et al., 2006 [41]	Brazil	Cross-sectional	Hospital Food Service	Diners + Food Service Workers	n.i.	n.i.	Self-Service	Lunch + dinner	2
Ofei et al., 2015 [42]	Denmark	Cross-sectional	Hospital Food Service	Diners	Self-managed	Plate	A la carte	Lunch + Supper	5 days
Pistorello et al., 2015 [43]	Brazil	Cross-sectional	Restaurant	Diners	n.i.	Plate	n.i.	Snacks	9
Pontes et al., 2022 [44]	Brazil	Cross-sectional	Restaurant	Diners	n.i.	Plate	Mixed	Lunch + dinner + Snacks	40
Quemelli et al., 2020 [45]	Brazil	Cross-sectional	Hospital Food Service	Food Service Workers	Outsourcing	Plate	Mixed	Lunch	2
Rabelo et al., 2016 [46]	Brazil	Cohort	Restaurant	Food Service Workers	Self-managed	Plate + Trays + lunchbox	Mixed	Lunch	4
Rodrigues et al., 2015 [47]	Brazil	Cross-sectional	Popular Food Service	n.i.	n.i.	Trays	n.i.	Lunch	13
Sabino et al., 2016 [48]	Brazil	Cross-sectional	Hospital Food Service	Diners + Food Service Workers	n.i.	Lunchbox	n.i.	n.i.	2
Santana et al., 2019 [49]	Brazil	Cross-sectional	Hospital Food Service	Food Service Workers	Outsourcing	Trays	Mixed	Lunch	1
Saputri et al., 2019 [50] *	Indonésia	Cross-sectional	University Restaurant	Diners + Food Service Workers	n.i.	n.i.	n.i.	n.i.	1
Scholz et al., 2019 [51] *	Brazil	Cross-sectional	Restaurant	Food Service Workers	Outsourcing	Trays	n.i.	Lunch	8
Silva et al., 2010 [52] *	Brazil	Cohort	Hospital Food Service	Food Service Workers	Self-managed	Trays	Self-Service	Lunch	8
Silvennoinen et al., 2015 [53] *	Finland	Case studies	Schools, day-care centers, University Restaurants, Restaurants. Cafes and petrol stations	n.i	n.i	Plate + Trays + lunch box	Mixed		
Strapazzon et al., 2016 [54] *	Brazil	Cross-sectional	n.i.	n.i.	n.i.	Trays	n.i.	n.i.	n.i.
Souza et al., 2022 [55]	Brazil	Cross-sectional	Hospital Food Service	Diners + Patient companion	Self-managed	Trays	Self-Service	Breakfast, morning snack, lunch, afternoon snack, dinner, and night snack	5 days
Thiagarajah et al., 2013 [56] *	USA	Cohort	University Restaurant	Diners + Food Service Workers	Self-managed	Plate	Self-Service	Lunch + dinner	17
Viana et al., 2016 [57]	Brazil	Cross-sectional	Hospital Food Service	Diners + Food Service Workers	n.i.	Plate	Self-Service	Lunch	1
Viana et al., 2017 [58]	Brazil	Cross-sectional	School Canteens	Food Service Workers	n.i.	Trays	Mixed	Lunch	½
Zandonadi et al., 2012 [59]	Brazil	Cross-sectional	Restaurant	Food Service Workers	n.i.	Plate	Mixed	Lunch	2
Zeineddine et al., 2021 [60] *	Lebanon	Ecologic study	Restaurant	Diners	Self-managed and outsourcing	Plate	Mixed	Dinner	76
Wang et al., 2017 [61] *	China	Survey study	Restaurant	Diners	Self-managed and outsourcing	Plate	Mixed	Dinner	52

Note: n.i. = not informed. Mixed is self-service and thermal counter. * Articles excluded from the meta-analysis.

**Table 2:** Food waste and total waste by type of food service, type of diners, type of distribution of meals, and distribution system.

	Number of Meals Offered (Absolute Number)	Plate Food Waste (kg) *	Weight of Studies	Plate Food Waste (%) by Subgroup Categories
**Food Service Type: Hospital Food Service**				
Chaves et al., 2019 [28]	152	7.76	2.9%	77.7%
Nonino Borges et al., 2006 [41]	650	24.0	3.8%	
Ofei et al., 2015 [42] (1)	142	8.62	2.7%	
Ofei et al., 2015 [42] (2)	114	6.1	2.6%	
Quemelli et al., 2020 [45]	184	7.15	3.2%	
Sabino et al., 2016 [48]	505	39.62	3.4%	
Santana et al., 2019 [49]	221	7.25	3.4%	
Souza et al., 2022 [55]	1472	7.7	4.0%	
Viana et al., 2016 [57]	67	3.0	2.2%	
Total	3507	111.2	28.1%	
**Food Service Type: School Canteens**				
Byker et al., 2014 [26]	304	45.3	2.6%	10.5%
Ilic et al., 2022 [33]	17,163	21	4.0%	
Liu et al., 2016 [34]	923	11.99	3.9%	
Lonska et al., 2022 [35]	7064	28.75	4.0%	
Viana et al., 2017 [58]	2329	13.74	4.0%	
Total	27,783	120.78	18.6%	
**Food Service Type: Restaurant (Commercial Food Service)**				
Augustini et al., 2008 [10]	4803	6.45	4.0%	7.3%
Carvalho et al., 2013 [11]	5849	6.87	4.0%	
Chang, 2021 [27]	360	0.93	4.0%	
Dagiliūtė and Musteikytė, 2019 [30]	174	14.74	2.6%	
Marais et al., 2017 [37]	586	16.90	3.8%	
Mello et al., 2011 [40]	3500	10.71	4.0%	
Pistorello et al., 2015 [43]	8389	30.71	4.0%	
Pontes et al., 2022 [44]	7997	6.67	4.0%	
Rabelo et al., 2016 [46]	440	9.45	3.8%	
Zandonadi et al., 2012 [59]	1646	4.39	4.0%	
Total	33,744	101.15	38.2%	
**Food Service Type: University Restaurant**				
Bicalho et al., 2013 [24]	193	10.67	3.0%	2.7%
Borges et al., 2019 [25]	1150	8.68	4.0%	
Coimbra et al., 2019 [29]	23,195	7.51	4.0%	
Total	24,538	26.86	11.0%	
**Food Service Type: Popular Food Service**				
Rodrigues et al., 2015 [47]	26,110	18.97	4.0%	1.7%
**Total overall**	**1,115,682**	**378.96**	**100.0%**	**100.0%**
**Diners type: Diners and food service workers**				
Borges et al., 2019 [25]	1150	8.68	4.5%	69.95%
Marais et al., 2017 [37]	586	16.9	4.3%	
Nonino Borges et al., 2006 [41]	650	24	4.2%	
Sabino et al., 2016 [48]	505	39.62	3.8%	
Viana et al., 2016 [57]	67	3.0	2.6%	
Total	2958	92,2	19.3%	
**Diners type: Food Service Workers**				
Augustini et al., 2008 [10]	4803	6.45	4.5%	9.41%
Bardini et al., 2014 [23]	1125	8.67	4.5%	
Carvalho et al., 2013 [11]	5849	6.87	4.5%	
Chaves et al., 2019 [28]	152	7.76	3.3%	
Quemelli et al., 2020 [45]	184	7.15	3.6%	
Rabelo et al., 2016 [46]	440	9.45	4.3%	
Santana et al., 2019 [49]	221	7.25	3.8%	
Total	12,774	53.6	28.4%	
**Diners type: Diners**				
Byker et al., 2014 [26]	304	45.3	3.0%	8.96%
Chang, 2021 [27]	360	0.93	4.5%	
Dagiliūtė and Musteikytė, 2019 [30]	174	14.74	2.9%	
Ilic et al., 2022 [33]	17,163	21	4.5%	
Liu et al., 2016 [34]	923	11.99	4.4%	
Lonska et al., 2022 [35]	7064	28.75	4.5%	
Ofei et al., 2015 [42] (1)	142	8.62	3.0%	
Ofei et al., 2015 [42] (2)	114	6.1	2.9%	
Pistorello et al., 2015 [43]	8389	30.71	4.5%	
Pontes et al., 2022 [44]	7997	6.67	4.5%	
Viana et al., 2017 [58]	2329	13.74	4.5%	
Zandonadi et al., 2012 [59]	1646	4.395	4.5%	
Total	46,605	186	47.8%	
**Diners type: Diners and patients’ companions**				
Souza et al., 2022 [55]	1472	7.7	4.5%	11.65%
**Total overall**	**63,809**	**339.77**	**100.0%**	**100.0%**
**Type of distribution: Lunch Box**				
Sabino et al., 2016 [48]	505	39.62	3.6%	72.82%
**Type of distribution: Lunch Box + Plate + Tray**				
Rabelo et al., 2016 [46]	440	9.45	4.0%	19.94%
**Type of distribution: Plate**				
Bardini et al., 2014 [23]	1125	8.67	4.2%	3.33%
Bicalho et al., 2013 [24]	193	10.67	3.2%	
Borges et al., 2019 [25]	1150	8.68	4.2%	
Byker et al., 2014 [26]	304	45.3	2.8%	
Chaves et al., 2019 [28]	152	7.76	3.1%	
Coimbra et al., 2019 [29]	23,195	7.51	4.2%	
Dagiliūtė and Musteikytė, 2019 [30]	174	14.74	2.8%	
Mello et al., 2011 [40]	3500	10.71	4.2%	
Ofei et al., 2015 [42] (1)	142	8.62	2.9%	
Ofei et al., 2015 [42] (2)	114	6.1	2.8%	
Pistorello et al., 2015 [43]	8389	30.71	4.2%	
Pontes et al., 2022 [44]	7997	6.67	4.2%	
Quemelli et al., 2020 [45]	184	7.15	3.4%	
Souza et al., 2022 [55]	1472	7.7	4.2%	
Viana et al., 2016 [57]	67	3.0	2.4%	
Zandonadi et al., 2012 [59]	1646	4395	4.2%	
Total	49,804	181.71	56.7%	
**Type of distribution: Tray**				
Aranha et al., 2018 [9]	152	8.73	3.0%	1.85%
Medeiros et al., 2014 [39]	896	9.96	4.1%	
Rodrigues et al., 2015 [47]	26,110	18.97	3.6%	
Santana et al., 2019 [49]	221	7.25	4.2%	
Viana et al., 2017 [58]	2329	13.74	4.2%	
Total	29,708	58.65	19.1%	
**Type of distribution: Plate + Tray**				
Augustini et al., 2008 [10]	4803	6.45	4.2%	2.13%
Ilic et al., 2022 [33]	17,163	21	4.2%	
Liu et al., 2016 [34]	923	11.99	4.1%	
Lonska et al., 2022 [35]	7064	28.75	4.2%	
Total	29,953	68.19	16.7%	
**Total overall**	**110,410**	**357.62**	**100.0%**	**100.0%**
**Management Mode: Self-Management**				
Borges et al., 2019 [25]	1150	8.68	14.8%	87.40%
Carvalho et al., 2013 [11]	5849	6.87	15.1%	
Marais et al., 2017 [37]	586	16.9	12.8%	
Mello et al., 2011 [40]	3500	10.71	15.1%	
Quemelli et al., 2020 [45]	184	7.15	8.4%	
Santana et al., 2019 [49]	221	7.25	9.7%	
Total	696	24.17	75.9%	
**Management Mode: Outsourcing**				
Ofei et al., 2015 [42] (1)	142	8.62	5.9%	12.60%
Ofei et al., 2015 [42] (2)	114	6.1	5.5%	
Rabelo et al., 2016 [46]	440	9.45	12.8%	
Total	11,490	57.56	24.1%	
**Total overall**	**12,186**	**81.73**	**100.0%**	**100.0%**
**Meal type: Lunch and Dinner**				
Augustini et al., 2008 [10]	4803	6.45	4.1%	5.24%
Borges et al., 2019 [25]	1150	8.68	4.1%	
Marais et al., 2017 [37]	586	16.9	3.8%	
Mello et al., 2011 [40]	3500	10.71	4.1%	
Nonino Borges et al., 2006 [41]	650	24	3.8%	
Pontes et al., 2022 [44]	7997	6.67	4.1%	
Total	18,686	66.74	24.0%	
**Meal type: Snacks**				
Pistorello et al., 2015 [43]	8389	30.71	4.1%	5.38%
**Meal type: Lunch**				
Aranha et al., 2018 [9]	152	8.73	2.5%	2.91%
Bardini et al., 2014 [23]	1125	8.67	4.1%	
Bicalho et al., 2013 [24]	193	10.67	2.7%	
Byker et al., 2014 [26]	304	45.3	2.3%	
Carvalho et al., 2013 [11]	5849	6.87	4.1%	
Chaves et al., 2019 [28]	152	7.76	2.6%	
Coimbra et al., 2019 [29]	23,195	7.51	4.1%	
Ilic et al., 2022 [33]	17,163	21	4.1%	
Liu et al., 2016 [34]	923	11.99	4.0%	
Lonska et al., 2022 [35]	7064	28.75	4.1%	
Medeiros et al., 2014 [39]	896	9.96	4.0%	
Ofei et al., 2015 [42] (1)	142	8.62	2.4%	
Quemelli et al., 2020 [45]	184	7.15	3.0%	
Rabelo et al., 2016 [46]	440	9.45	3.8%	
Rodrigues et al., 2015 [47]	26,110	18.97	4.1%	
Santana et al., 2019 [49]	221	7.25	3.3%	
Viana et al., 2016 [57]	67	3.0	1.9%	
Viana et al., 2017 [58]	2329	13.74	4.1%	
Zandonadi et al., 2012 [59]	1646	4395	4.1%	
Total	62,559	124.12	65.5%	
**Meal type: Supper**				
Ofei et al., 2015 [42] (2)	114	6.1	2.2%	77.87%
Meal type: Small and large meals **				
Souza et al., 2022	1472	7.7	4.1%	7.56%
**Total overall**	**116,816**	**351.03**	**100.0%**	**100.0%**
**Distribution system: Self-Service**				
Bardini et al., 2014 [23]	1125	8.67	4.4%	61.42%
Bicalho et al., 2013 [24]	193	10.67	3.2%	
Byker et al., 2014 [26]	30.10	45.3	2.8%	
Carvalho et al., 2013 [11]	5849	6.87	4.4%	
Chaves et al., 2019 [28]	152	7.76	3.1%	
Nonino Borges et al., 2006 [41]	650	24	4.1%	
Viana et al., 2016 [57]	67	3.0	2.4%	
Total	13,143	112.72	24.5%	
**Distribution system: *A la carte***				
Ilic et al., 2022 [33]	27.12	21	4.4%	22.14%
Liu et al., 2016 [34]	11.7	11.99	4.3%	
Lonska et al., 2022 [35]	4.5	28.75	4.4%	
Ofei et al., 2015 [42] (1)	21.5	8.62	2.9%	
Ofei et al., 2015 [42] (2)	23.4	6.1	2.8%	
Souza et al., 2022 [55]	11.1	7.7	4.4%	
Total	26,878	84.16	23.2%	
Distribution system: Mixed ***				
Aranha et al., 2018 [9]	152	8.73	3.0%	16.42%
Augustini et al., 2008 [10]	23.2	6.45	4.4%	
Borges et al., 2019 [25]	1150	8.68	4.4%	
Chang, 2021 [27]	25.12	0.93	4.4%	
Coimbra et al., 2019 [29]	23,195	7.51	4.4%	
Dagiliūtė and Musteikytė, 2019 [30]	22.6	14.74	2.8%	
Mello et al., 2011 [40]	31.7	10.71	4.4%	
Pontes et al., 2022 [44]	22.11	6.67	4.4%	
Quemelli et al., 2020 [45]	184	7.15	3.5%	
Rabelo et al., 2016 [46]	440	9.45	4.2%	
Santana et al., 2019 [49]	221	7.25	3.7%	
Viana et al., 2017 [58]	2329	13.74	4.4%	
Zandonadi et al., 2012 [59]	1646	4.39	4.4%	
Total	41,348	93.28	52.3%	
**Total overall**	**81,369**	**290.16**	**100.0%**	**100.0%**

Note: * Weight of plate food waste is total weight for the amount of people; ** Breakfast, snacks, lunch, dinner, and supper. Small meal is snacks + supper. Large dinner is breakfast + lunch + dinner. *** Mixed is a self-service and thermal counter.

**Table 3:** Number of meals offered, plate food waste per capita (kg and %) by type of food service, type of diners, type of distribution, type of meal, and distribution system.

	Number of Meals Offered (Absolute Number)	Per Capita (kg) Plate Food Waste in the Period of the Study	Plate Food Waste per Capita (%)
**Food Service Type**			
University Restaurant	37,788	9.92	0.03
Hospital Food Service	4222	0.95	0.02
Restaurant	54,685	4.00	0.01
School Canteens	20,415	0.08	0.00
Popular Food Service	26,110	0.09	0.00
**Type of diners**			
Food Service Workers	25,175	2.37	0.01
Diners and Food Service Workers	16,208	0.57	0.00
Diners	38,621	0.23	0.00
Diners and Companies	1472	0.03	0.00
**Type of distribution**			
Lunchbox	505	0.17	0.03
Plate + Tray	22,889	0.74	0.00
Plate	69,546	3.92	0.01
Lunch Box + Plate + Tray	440	0.06	0.01
Tray	40,498	1.01	0.00
**Distribution Modality**			
Self-Management	15,005	3.77	0.03
Outsourcing	16,078	0.54	0.00
**Type of Meal**			
Lunch	102,599	5.52	0.01
Lunch and Dinner	27,866	1.11	0.00
Snacks	8389	0.07	0.00
Small and Large meals *	1472	0.03	0.00
**Distribution System**			
Mixed **	45,335	4.05	0.01
Self-Service	28,246	1.55	0.01
Assisted service	19,558	0.03	0.00

Note: * Breakfast, snacks, lunch, dinner, and supper. Small meal is snacks + supper. Large dinner is breakfast + lunch + dinner. ** Mixed is self-service and thermal counter.

## Supplementary Materials

The following supporting information can be downloaded at: https://www.mdpi.com/article/10.3390/nu16101429/s1, Table S1: Indexers used to select publications; Table S2: JBI Critical Appraisal Checklist (Risk of Bias); Table S3: Full-text excluded articles and reasons.
