# Supplementary material for: Plate Food Waste in Food Services: A Systematic Review and Meta-Analysis
Source: Nutrients. 2024 May 9;16(10):1429. doi: 10.3390/nu16101429 (PMC11123872; doi:10.3390/nu16101429)
Supplement: Supplementary file 1 [file nutrients-16-01429-s001.zip › nutrients-3001650-supplementary.pdf]

nutrients MDPI

Supplementary Materials

Table S1. Indexers used to select publications

**PubMed 1269**	("food services"[MeSH Terms] OR ("food services"[MeSH Terms] OR ("food"[All Fields] AND "services"[All Fields]) OR "food services"[All Fields] OR ("services"[All Fields] AND "food"[All Fields]) OR "services food"[All Fields]) OR ("food services"[MeSH Terms] OR ("food"[All Fields] AND "services"[All Fields]) OR "food services"[All Fields] OR ("food"[All Fields] AND "service"[All Fields]) OR "food service"[All Fields]) OR ("food services"[MeSH Terms] OR ("food"[All Fields] AND "services"[All Fields]) OR "food services"[All Fields] OR ("service"[All Fields] AND "food"[All Fields]) OR "service food"[All Fields]) OR ("food services"[MeSH Terms] OR ("food"[All Fields] AND "services"[All Fields]) OR "food services"[All Fields] OR ("meal"[All Fields] AND "wheels"[All Fields])) OR ("food services"[MeSH Terms] OR ("food"[All Fields] AND "services"[All Fields]) OR "food services"[All Fields] OR ("meals"[All Fields] AND "wheels"[All Fields]) OR "meals on wheels"[All Fields]) OR "food service, hospital"[MeSH Terms] OR ("food service, hospital"[MeSH Terms] OR ("food"[All Fields] AND "service"[All Fields] AND "hospital"[All Fields]) OR "hospital food service"[All Fields] OR ("service"[All Fields] AND "hospital"[All Fields] AND "food"[All Fields])) OR ("food service, hospital"[MeSH Terms] OR ("food"[All Fields] AND "service"[All Fields] AND "hospital"[All Fields]) OR "hospital food service"[All Fields] OR ("food"[All Fields] AND "services"[All Fields] AND "hospital"[All Fields])) OR ("food service, hospital"[MeSH Terms] OR ("food"[All Fields] AND "service"[All Fields] AND "hospital"[All Fields]) OR "hospital food service"[All Fields] OR ("hospital"[All Fields] AND "food"[All Fields] AND "services"[All Fields]) OR "hospital food services"[All Fields]) OR ("food service, hospital"[MeSH Terms] OR ("food"[All Fields] AND "service"[All Fields] AND "hospital"[All Fields]) OR "hospital food service"[All Fields] OR ("services"[All Fields] AND "hospital"[All Fields] AND "food"[All Fields])) OR ("food service, hospital"[MeSH Terms] OR ("food"[All Fields] AND "service"[All Fields] AND "hospital"[All Fields]) OR "hospital food service"[All Fields] OR ("hospital"[All Fields] AND "food"[All Fields] AND "service"[All Fields])) OR "restaurants"[MeSH Terms] OR ("restaurant s"[All Fields] OR "restaurants"[MeSH Terms] OR "restaurants"[All Fields] OR "restaurant"[All Fields])) AND ("Sustainable practices"[All Fields] OR "Sustainability"[All Fields] OR "Environmental sustainability"[All Fields])
**LILACS (by BVS) 1767**	"Serviços de Alimentação" OR " Food Services" OR "Servicios de Alimentación" OR "Services alimentaires" OR "Serviço Hospitalar de Nutrição" OR "Food Service, Hospital" OR "Servicio de Alimentación en Hospital" OR " Restaurantes" OR "Restaurants" AND (db:("LILACS"))
**Embase 9687**	'environmental sustainability'/exp OR 'envinonmental sustainability' AND 'sustainability'/exp OR sustainability AND 'food services'/exp OR 'food services'/syn AND 'restaurant'/exp OR 'restaurant'/syn AND 'hospital food service'/exp OR 'hospital food service'/syn
**Other databases : « IBECS" OR "BINACIS"**	"Serviços de Alimentação" OR " Food Services" OR "Servicios de Alimentación" OR "Services alimentaires" OR "Serviço Hospitalar de Nutrição" OR "Food Service, Hospital" OR "Servicio de Alimentación en Hospital" OR " Restaurantes" OR "Restaurants" AND (db:("IBECS" OR

**OR "BDENF" OR "CUMED" OR "BDNPAR" OR "ARGMSAL" OR "SDG" 410**	"BINACIS" OR "BDENF" OR "CUMED" OR "BDNPAR" OR "ARGMSAL" OR "SDG"))

Table S2. JBI Critical Appraisal Checklist (Risk of Bias)

Author (year)	1	2	3	4	5	6	7	8	9
Aranha et al., 2018 [1]	N	U	Y	N	Y	Y	Y	Y	NA
Augustini et al., 2008 [2]	Y	Y	Y	Y	Y	Y	Y	Y	NA
Barbosa et al., 2021 [3]	Y	Y	Y	Y	Y	Y	Y	Y	NA
Bardini et al., 2014 [4]	Y	Y	Y	N	Y	N	N	Y	NA
Bicalho et al., 2013 [5]	N	U	Y	N	Y	Y	Y	Y	NA
Borges et al., 2019 [6]	Y	Y	Y	Y	Y	Y	Y	Y	NA
Byker et al., 2014 [7]	Y	Y	Y	Y	Y	Y	Y	Y	NA
Carvalho et al., 2013 [8]	Y	Y	Y	Y	Y	Y	Y	Y	NA
Chang, 2021 [9]	Y	Y	Y	Y	Y	N	N	Y	NA
Chaves et al., 2019 [10]	Y	Y	Y	Y	Y	Y	Y	Y	NA
Coimbra et al., 2019 [11]	N	U	Y	N	Y	U	U	Y	NA
Dagiliūtė & Musteikytė, 2019 [12]	Y	Y	Y	Y	Y	Y	N	Y	NA
Delazeri et al., 2015 [13]	N	U	Y	N	Y	Y	Y	Y	NA
Galego et al., 2014 [14]	Y	Y	Y	N	Y	N	N	Y	NA
Ilic et al., 2022 [15]	Y	Y	Y	Y	Y	Y	Y	Y	NA
Liu et al., 2016 [16]	Y	Y	Y	Y	Y	Y	Y	Y	NA
Lonska et al., 2022 [17]	Y	Y	Y	Y	Y	Y	Y	Y	NA
Machado et al., 2014 [18]	Y	Y	Y	Y	Y	Y	Y	Y	NA
Marais et al., 2017 [19]	Y	Y	Y	Y	Y	Y	Y	Y	NA
Matzemacher et al., 2020 [20]	N	U	Y	N	Y	N	N	Y	NA
Medeiros et al., 2014 [21]	N	U	Y	N	Y	Y	Y	Y	NA
Mello et al., 2011 [22]	N	U	Y	N	Y	Y	Y	Y	NA
Nonino Borges et al., 2006 [23]	Y	Y	Y	Y	Y	N	N	Y	NA
Ofei et al., 2015 [24]	Y	Y	Y	Y	Y	Y	Y	Y	NA
Pistorello et al., 2015 [25]	Y	Y	Y	Y	Y	N	N	Y	NA
Pontes et al., 2022 [26]	Y	Y	Y	Y	Y	Y	Y	Y	NA
Quemelli et al., 2020 [27]	Y	Y	Y	Y	Y	Y	Y	Y	NA
Rabelo et al., 2016 [28]	Y	Y	Y	Y	Y	Y	Y	Y	NA
Rodrigues et al., 2015 [29]	N	U	Y	N	Y	Y	Y	Y	NA
Sabino et al., 2016 [30]	Y	Y	Y	Y	Y	Y	Y	Y	NA
Santana et al., 2019 [31]	Y	Y	Y	Y	Y	Y	Y	Y	NA
Saputri et al., 2019 [32]	Y	Y	Y	N	Y	N	N	Y	NA
Scholz et al., 2019 [33]	Y	Y	Y	Y	Y	Y	Y	Y	NA
Silva et al., 2010 [34]	Y	Y	Y	Y	Y	Y	Y	Y	NA
Silvennoinen et al., 2015 [35]	Y	Y	Y	Y	Y	N	N	Y	NA
Strapazzon et al., 2016 [36]	N	U	Y	N	Y	N	N	Y	NA

Souza et al., 2022 [37]	Y	Y	Y	Y	Y	Y	Y	Y	NA
Thiagarajah et al., 2013 [38]	Y	Y	Y	Y	Y	N	N	Y	NA
Viana et al., 2016 [39]	Y	Y	Y	Y	Y	Y	Y	Y	NA
Viana et al., 2017 [40]	Y	Y	Y	Y	Y	Y	Y	Y	NA
Zandonadi et al., 2012 [41]	Y	Y	Y	Y	Y	Y	Y	Y	NA
Zeineddine et al., 2021 [42]	Y	Y	Y	Y	Y	Y	Y	Y	NA
Wang et al., 2017 [43]	Y	Y	Y	Y	Y	Y	Y	Y	NA
**Total**	**Yes** : 75.19%		**No:** 21.96%		**Unclear:** 2.84%		**Not Applicable:** 11.11%		

Questions: **1**. Was the sample frame appropriate to address the target population?; **2.** Were study participants sampled in an appropriate way?; **3.** Was the sample size adequate?; **4.** Were the study subjects and the setting described in detail?; **5.** Was the data analysis conducted with sufficient coverage of the identified sample?; **6.** Were valid methods used for the identification of the condition?; **7.** Was the condition measured in a standard, reliable way for all participants?; **8.** Was there appropriate statistical analysis?; **9.**Was the response rate adequate, and if not, was the low response rate managed appropriately?

Table S3. Full-text excluded articles and reasons

Author (year)	Reference	Exclusion Motif
Alamar et al., 2017	[44]	1
Anwar et al., 2012	[45]	4
Araújo et al., 2023	[46]	1
*Ariefuddin et al., 2009	[47]	4
Armadita et al., 2019	[48]	3
Armendro, 2021	[49]	1
Baldwin et al., 2010	[50]	1
Balzaretti et al., 2018	[51]	1
*Barton et al., 2000	[52]	3
Bassin et al., 2019	[53]	1
*Boschini et al., 2018	[54]	6
*Boschini et al., 2020	[55]	6
Boutler and Blanchard, 2021	[56]	1
Carvalho et al., 2018	[57]	1
Castro, 2011	[58]	1
*Cerrah and Yigitoglu, 2022	[59]	3
Cook Hoons et al., 2022	[60]	1
Corrêa et al., 2006	[61]	2
Cristobal et al., 2017	[62]	1
Derqui and Fernandez, 2017	[63]	1
Di Pierro et al., 2023	[64]	1
*Diana et al., 2022	[65]	8
*Dias-Ferreira et al., 2015	[66]	4
*Djamaluddin et al., 2005	[67]	3
Dogdu et al., 2024	[68]	1
Elnakib et al., 2021	[69]	1
Fassina et al., 2019	[70]	1
*Fatkhurohman et al., 2017	[71]	4
Hannibal and Vedlitz, 2018	[72]	1
Heller and Silva, 2015	[73]	1
*Heryawanti et al., 2004	[74]	4
Hidalgo-Víquez and Peña Vásquez, 2021	[75]	1
*Hopkins et al., 2023	[76]	3

*Iqbal et al., 2016	[77]	4
*Irawati et al., 2010	[78]	3
Ismail et al., 2019	[79]	1
Jia et al., 2023	[80]	1
Jones et al., 2012	[81]	1
Khalid et al., 2023	[82]	1
*Kodors et al., 2022	[83]	3
Lins et al., 2021	[84]	1
*Longo-Silva et al., 2013	[85]	3
Love et al., 2023	[86]	1
Lu et al., 2023	[87]	1
*Mardianingsih et al., 2020	[88]	4
Martin-Rios et al., 2022	[89]	1
Martindale and Schiebel, 2017	[90]	1
Mathisen and Johansen, 2022	[91]	1
Medeiros et al., 2022	[92]	1
Monteiro et al., 2014	[93]	1
Musicus et al., 2019	[94]	1
Ñunque et al., 2015	[95]	1
O'Connor et al., 2022	[96]	1
Ögel et al., 2023	[97]	2
Osei-Owusu et al., 2023	[98]	1
Palmer et al., 2021	[99]	1
*Partearroyo et al., 2020	[100]	5
Pereira et al., 2019	[101]	1
Pettinger et al., 2023	[102]	1
Pinard et al., 2014	[103]	1
Prescott et al., 2019	[104]	1
Principato et al., 2018	[105]	1
*Razalli et al., 2021	[106]	4
*Rochmah, 2020	[107]	4
*Roe et al., 2018	[108]	5
*Roe et al., 2020	[109]	5
*Schwartz et al., 2015	[110]	3
Silva et al., 2013	[111]	1
Strasburg et al., 2023	[112]	1
Strotmann et al., 2022	[113]	1
*Syauqiyatullah et al., 2020	[114]	4
Tanuwijaya et al., 2018	[115]	4
Tenser et al., 2007	[116]	1
Whitehair et al., 2013	[117]	1
*Wilkie et al., 2015	[118]	7
Yazdankhah et al., 2020	[119]	1

Legend – Exclusion criteria: 1 - Does not include data on intake (n= 47); 2 - No access, authors contacted without reply (n= 2); 3 - Not had % plaste waste and number of meals and only food groups (n= 9); 4 - Texture or type of modified diet (n= 11); 5 - Not specific food service < free-living conditions> (n= 3); 6 - Outcome not measure (n= 2); 7 - School kitchen or brought from home without separation (n= 1); 8 - Systematic review (n=1) *Excluded studies for hand search..

References

1. Aranha, F.Q.; Flora Silva Gustavo, A. Avaliação Do Desperdício De Alimentos Em Uma Unidade De Alimentação E Nutrição Na Cidade De Botucatu.
2. Augustini, V.C. de M.; K.P.; T.T.C.; D. almeida, F.Q.A. Avaliação Do Índice De Resto-Ingesta E Sobras Em Unidade De Alimentação E Nutrição (UAN) De Uma Empresa Metalúrgica Na Cidade De Piracicaba/SP. *Rev. Simbio-Logias* **2008**.
3. Barbosa, A.K. da S.; Lima, M.F.; Lima, W.L. Avaliação Do Resto E Ingesta de Refeições Em Um Restaurante de Empresa Privada. *Higiene Alimentar* **2021**, 35, e1027–e1027, doi:https://doi.org/10.37585/HA2021.01avaliacao.
4. Bardini, M.M.V.; Cruz, A. Determinação Do Índice de Resto-Ingestão Em Unidade de Alimentação e Nutriçao Do Município de Tubarão, SC. *Higiene Alimentar* **2014**, 53–57.
5. Bicalho, A.H.; Lima, V.O.B. Impacto de Uma Intervenção Para Redução Do Desperdício Em Uma Unidade de Alimentação e Nutrição. *Nutrire* **2013**, *38*, 269–277, doi:10.4322/nutrire.2013.025.
6. Borges, M.P.; Souza, L.H.R.; De Pinho, S.; De Pinho, L. Impacto de Uma Campanha Para Redução de Desperdício de Alimentos Em Um Restaurante Universitário. *Engenharia Sanitaria e Ambiental* **2019**, *24*, 843–848, doi:10.1590/S1413-41522019187411.
7. Byker, C.J.; Farris, A.R.; Marcenelle, M.; Davis, G.C.; Serrano, E.L. Food Waste in a School Nutrition Program After Implementation of New Lunch Program Guidelines. *J Nutr Educ Behav* **2014**, *46*, 406–411, doi:10.1016/j.jneb.2014.03.009.
8. Carvalho, E.M. de; Fonseca, C.S.; Castro, L.C.V.; Costa, A.C. Avaliação Do Índice de Resto-Ingestão e Sobras Em Uma Unidade Produtora de Refeição (UPR). *Higiene Alimentar* **2013**, *27*, 19–22.
9. Chang, Y.Y.C. All You Can Eat or All You Can Waste? Effects of Alternate Serving Styles and Inducements on Food Waste in Buffet Restaurants. *Current Issues in Tourism* **2022**, *25*, 727–744, doi:10.1080/13683500.2020.1870939.
10. Chaves, V.S.; Carolina, C.; Machado, B.; De, V.; Abreu, S. Índice de Resto Ingestão Antes e Após Campanha de Conscientização de Comensais. *Revista EVS - Revista de Ciências Ambientais e Saúde* **2019**, *46*, 1–7, doi:10.18224/evs.v45i1.6185.
11. Coimbra, A.L.Q.; Silva, L.K.R.; Lacerda, R.S.; Chagas, G.V.; Trindade, S.N.C. Índice de Resto-Ingestão e Avaliação Qualitativa Das Preparações Do Cardápio de Um Restaurante Universitário Do Município de Barreiras-BA / Rest Index-Ingestion and Qualitative Evaluation of the Preparations of the Menu of a University Restaurant in Barreiras-BA. *Higiene alimentar* **2019**, *33*, 398–402.
12. Dagiliūtė, R.; Musteikytė, A. Food Waste Generation: Restaurant Data and Consumer Attitudes. *Environmental Research, Engineering and Management* **2019**, *75*, 7–14, doi:10.5755/J01.EREM.75.2.22995.
13. Delazeri, P.C.; Batisti, S.L.; Silva, A.B.G. Avaliação E Campanha Para Diminuição Do Resto Em Uma Unidade De Alimentação E Nutrição De Uma Empresa Do Vale Do Taquari. RS. *Higiene Alimentar* **2015**, *29*, 37–43.
14. Galego, B.V.; Russo, C.B.; Moura, P.N. Síntese Avaliação Do Índice De Desperdício Do Refeitório De Uma UAN Do Município De Guarapuava -PR. *Higiene Alimentar* **2014**, *28*, 202–204.
15. Ilić, A.; Bituh, M.; Brečić, R.; Colić Barić, I. Relationship Between Plate Waste and Food Preferences Among Primary School Students Aged 7–10 Years. *J Nutr Educ Behav* **2022**, *54*, 844–852, doi:10.1016/J.JNEB.2022.04.003.

16. Liu, Y.; Cheng, S.; Liu, X.; Cao, X.; Xue, L.; Liu, G. Plate Waste in School Lunch Programs in Beijing, China. *Sustainability 2016, Vol. 8, Page 1288* **2016**, *8*, 1288, doi:10.3390/SU8121288.
17. Lonska, J.; Zvaigzne, A.; Kotane, I.; Silicka, I.; Litavniece, L.; Kodors, S.; Deksne, J.; Vonoga, A. Plate Waste in School Catering in Rezekne, Latvia. *Sustainability 2022, Vol. 14, Page 4046* **2022**, *14*, 4046, doi:10.3390/SU14074046.
18. Machado, C.C.B.; Mendes, C.K.; Souza, P.G.; Martins, K.S.R.; Silva, K.C.C. Avaliação Do Índice de Resto Ingesta de Uma Unidade de Alimentação e Nutrição Institucional de Anápolis-GO. *Ensaios e Ciência C Biológicas Agrárias e da Saúde* **2012**, *16*.
19. Marais, M.L.; Smit, Y.; Koen, N.; Lötze, E. Are the Attitudes and Practices of Foodservice Managers, Catering Personnel and Students Contributing to Excessive Food Wastage at Stellenbosch University? *South African Journal of Clinical Nutrition* **2017**, *30*, 15–22, doi:10.1080/16070658.2017.1267348.
20. Matzembacher, D.E.; Brancoli, P.; Moltene Maia, L.; Eriksson, M. Consumer's Food Waste in Different Restaurants Configuration: A Comparison between Different Levels of Incentive and Interaction. *Waste Management* **2020**, *114*, 263–273, doi:10.1016/J.WASMAN.2020.07.014.
21. Medeiros, L.B.; Saccol, A.L.F. Avaliação Do Índice De Resto E Sobras Em Serviços De Alimentação. *Higiene Alimentar* **2014**, *28*, 64–68.
22. Mello, A.G. de; Back, F. da S.; Baratta, R.; Pires, L.A.; Colares, L.G.T. Avaliação Do Desperdício de Alimentos Em Unidade de Alimentação e Nutrição Localizada Em Um Clube Da Cidade Do Rio de Janeiro. *Higiene Alimentar* **2011**, 33–39.
23. Nonino-Borges, C.B.; Rabito, E.I.; Da Silva, K.; Ferraz, C.A.; Chiarello, P.G.; Dos Santos, J.S.; Marchini, J.S. Desperdício de Alimentos Intra-Hospitalar. *Revista de Nutrição* **2006**, *19*, 349–356, doi:10.1590/S1415-52732006000300006.
24. Ofei, K.T.; Holst, M.; Rasmussen, H.H.; Mikkelsen, B.E. Effect of Meal Portion Size Choice on Plate Waste Generation among Patients with Different Nutritional Status. An Investigation Using Dietary Intake Monitoring System (DIMS). *Appetite* **2015**, *91*, 157–164, doi:10.1016/J.APPET.2015.04.043.
25. Pistorello, J.; Conto, S.M. de; Zaro, M. Geração de Resíduos Sólidos Em Um Restaurante de Um Hotel Da Serra Gaúcha, Rio Grande Do Sul, Brasil. *Engenharia Sanitaria e Ambiental* **2015**, *20*, 337–346, doi:10.1590/S1413-41522015020000133231.
26. Pontes, T.O.; da Silva César, A.; Conejero, M.A.; Rodrigues Deliberador, L.; Otávio Batalha, M. Food Waste Measurement in a Chain of Industrial Restaurants in Brazil. *J Clean Prod* **2022**, *369*, 133351, doi:10.1016/J.JCLEPRO.2022.133351.
27. Quemelli, C.A.; Nogueira, G.B. Avaliação Da Sobra E Do Resto Ingesta Como Estratégia Na Redução Do Desperdício De Alimentos. *Saber Científico* **2020**, *9*.
28. Rebelo, N.M.L.; Alves, T.C.U. Avaliação Do Percentual de Resto-Ingestão e Sobra Alimentar Em Uma Unidade de Alimentação e Nutrição Institucional. *Revista Brasileira de Tecnologia Agroindustrial* **2016**, *10*, 2039–2025.
29. Rodrigues, A.N.; Mendonça, X.M.F.D.; Nascimento, F.C.A. Estudo Do Desperdício de Alimentos Em Um Restaurante Popular de Belém - PA: Foco Na Sustentabilidade e Qualidade de Vida. *Higiene alimentar* **2015**, *29*, 133–137.
30. Sabino, J.B.; Brasileiro, N.P.M.; Souza, L.T. Pesquisa de Resto-Ingesta Em Uma Unidade de Alimentação e Nutrição Hospitalar de Teófilo Otoni - MG. *Higiene alimentar* **2016**, *30*, 24–27.

31. Santana, K.L.; Fernandes, C.E.; Oliveira, L.G.B.; Santos, V.V.; Guerra, J.M.C. Análise Do Índice De Resto-Ingesta E De Sobra Suja Em Uma UAN Hospitalar De Recife - PE. *Higiene Alimentar* **2019**, *33*, 133–137.
32. Saputri, E.M.; Tangsuphoom, N.; Rojroongwasinkul, N. Nutritional Impact of Plate Waste in University Canteens: An Assessment at Mulawarman University. *Abstracts Ann Nutr Metab* **2019**, *75*, 424.
33. Scholz, F.; Michele Dutra Rosolen, L.; Fernanda Scherer Adami, L.; Patricia Fassina, L.; Correspondência para, L. Avaliação Do Resto-Ingesta Antes e Durante Uma Campanha de Conscientização Contra o Desperdício de Alimentos. *Nutrivisa - Revista de Nutrição e Vigilância em Saúde* **2019**, *6*, 1–9, doi:10.59171/NUTRIVISA-2019V6E9284.
34. Silva, A.M.D.; Silva, C.P.; Pessina, E.L. Avaliação Do Índice De Resto Ingesta Após Campanha De Conscientização Dos Clientes Contra O Desperdício De Alimentos Em Um Serviço De Alimentação Hospitalar. *Revista Simbio-Logias* **2010**, 1–3.
35. Silvennoinen, K.; Heikkilä, L.; Katajajuuri, J.M.; Reinikainen, A. Food Waste Volume and Origin: Case Studies in the Finnish Food Service Sector. *Waste Management* **2015**, *46*, 140–145, doi:10.1016/J.WASMAN.2015.09.010.
36. Strapazzon, J.; Aralde, Q.M.; Anjos, M.B. dos; Cozer, M.; França, V.F. Sobras e Resto Ingesta: Uma Avaliação Do Desperdício. *Nutrição Brasil* **2015**, *14*, doi:10.33233/NB.V14I3.216.
37. Souza, B.J.; Monteiro, C.C.; Costa, V.P.G.; Borges, T.A.M.; Oliva, P.A.B.F. Food Consumption and Plate Waste Study in a Public Hospital Food Service in Natal, RN, Brazil. *GSC Advanced Research and Reviews* **2022**, *11*, 056–065, doi:10.30574/gscarr.2022.11.3.0153.
38. Thiagarajah, K.; Getty, V.M. Impact on Plate Waste of Switching from a Tray to a Trayless Delivery System in a University Dining Hall and Employee Response to the Switch. *J Acad Nutr Diet* **2013**, *113*, 141–145, doi:10.1016/j.jand.2012.07.004.
39. Viana, K.L.S.; Souza, A.L.M. de Avaliação Do Indice De Resto Ingestão. Antes E Durante Uma Campanha Educativa. Em Unidade De Alimentação E Nutrição (Uan). Porto Velho –RO. *Connection Line - Revista Eletrônica Do UNIVAG* **2016**, *0*, doi:10.18312/CONNECTIONLINE.V0I14.322.
40. Viana, R.M.; Ferreira, L.C. Avaliação Do Desperdício De Alimentos Em Unidade De Alimentação E Nutrição Cidade De Januária. MG. *Higiene Alimentar* **2017**, *31*.
41. Zandonadi, H.S.; Maurício, A.A. Avaliação Do Índice De Resto-Ingesta. De Refeições Consumidas Por Trabalhadores Da Construção Civil No Município De Cuiabá. *Higiene alimentar* **2012**, *26*, 64–70.
42. Zeineddine, M.; Kharroubi, S.; Chalak, A.; Hassan, H.; Abiad, M.G. Post-Consumer Food Waste Generation While Dining out: A Close-up View. *Plos One* **2021**, *16*, e0251947, doi:10.1371/JOURNAL.PONE.0251947.
43. Wang, L. en; Liu, G.; Liu, X.; Liu, Y.; Gao, J.; Zhou, B.; Gao, S.; Cheng, S. The Weight of Unfinished Plate: A Survey Based Characterization of Restaurant Food Waste in Chinese Cities. *Waste Management* **2017**, *66*, 3–12, doi:10.1016/J.WASMAN.2017.04.007.
44. Alamar, M. del C.; Falagán, N.; Aktas, E.; Terry, L.A. Minimising Food Waste: A Call for Multidisciplinary Research. *Journal of the Science of Food and Agriculture* **2017**, *98*, 8–11, doi:https://doi.org/10.1002/jsfa.8708.
45. Anwar, I.; Herianandita, E.; Ruslita, I. Evaluasi Sistem Penyelenggaraan Makanan Lunak Dan Analisis Sisa Makanan Lunak Di Beberapa Rumah Sakit Di Dki Jakarta, Tahun 2011. *Gizi Indonesia* **2012**, *35*, doi:https://doi.org/10.36457/gizindo.v35i2.126.

46. Araújo, F.D.; Clemente, H.A.; Lira, N. de C.C.; Moura, L.D.S. de; Oliveira, V.T. de L. Avaliação Qualitativa Das Preparações Servidas Em Um Restaurante Popular E Os Impactos Do Cenário Pandêmico Da COVID-19. *Revista Ciência Plural* **2023**, *9*, 1–18, doi:https://doi.org/10.21680/2446-7286.2023v9n1id29294.
47. Ariefuddin, M.A.; Kuntjoro, T.; Prawiningdyah, Y. Analisis Sisa Makanan Lunak Rumah Sakit Pada Penyelenggaraan Makanan Dengan Sistem Outsourcing Di RSUD Gunung Jati Cirebon. *Jurnal Gizi Klinik Indonesia* **2009**, *5*, 133, doi:https://doi.org/10.22146/ijcn.17570.
48. Armadita, P.D.J.; Primadona, S.; Mahmudiono, T. Vegetables Taste, Appearance, and Waste in Pediatric Patients at Dr. Ramelan Naval Hospital Surabaya. *Media Gizi Indonesia* **2019**, *14*, 66, doi:https://doi.org/10.20473/mgi.v14i1.66-74.
49. Armendro, J.N. Valorização de Resíduos Orgânicos E Redução de Desperdício No Ramo Industrial de Fornecimento de Suprimentos Alimentares Para Restaurantes Do Tipo \"Fast-Food\." Dissertação De Mestrado, Universidade de São Paulo - USP, 2021.
50. Baldwin, C.; Wilberforce, N.; Kapur, A. Restaurant and Food Service Life Cycle Assessment and Development of a Sustainability Standard. *The International Journal of Life Cycle Assessment* **2010**, *16*, 40–49, doi:https://doi.org/10.1007/s11367-010-0234-x.
51. Balzaretti, C.M.; Ventura, V.; Ratti, S.; Ferrazzi, G.; Spallina, A.; Carruba, M.O.; Castrica, M. Improving the Overall Sustainability of the School Meal Chain: The Role of Portion Sizes. *Eating and Weight Disorders - Studies on Anorexia, Bulimia and Obesity* **2018**, *25*, 107–116, doi:https://doi.org/10.1007/s40519-018-0524-z.
52. Barton, A.D.; Beigg, C.L.; Macdonald, I.A.; Allison, S.P. High Food Wastage and Low Nutritional Intakes in Hospital Patients. *Clinical Nutrition* **2000**, *19*, 445–449, doi:https://doi.org/10.1054/clnu.2000.0150.
53. Bassin, T.A.; Medeiros, M. de Q.; Oliveira, F. dos S.F.D.D. Avaliação Do Controle de Fator de Correção de Hortifrutis Em Uma Unidade de Alimentação E Nutrição Na Baixada Fluminense Do Rio de Janeiro. *Higiene Alimentar* **2019**, *33*, 261–265.
54. Boschini, M.; Falasconi, L.; Giordano, C.; Alboni, F. Food Waste in School Canteens: A Reference Methodology for Large-Scale Studies. *Journal of Cleaner Production* **2018**, *182*, 1024–1032, doi:https://doi.org/10.1016/j.jclepro.2018.02.040.
55. Boschini, M.; Falasconi, L.; Cicatiello, C.; Franco, S. Why the Waste? A Large-Scale Study on the Causes of Food Waste at School Canteens. *Journal of Cleaner Production* **2020**, *246*, 118994, doi:https://doi.org/10.1016/j.jclepro.2019.118994.
56. Boulter, A.; Blanchard, L. The Attitudes and Experience of Workplace Catering Staff Regarding Food Sustainability: A Qualitative Study. *The London School of Hygiene & Tropical Medicine* **2021**.
57. Carvalho, L.R. de; Rennó, P.B.; Lourenço, M.S.; Medeiros, M. das G.G.A.; Mazoto, M.L. Práticas Educativas de Gestão Ambiental Nos Serviços de Alimentação Permissionários Dos Campi de Universidade Pública Do Estado Do Rio de Janeiro. *Higiene Alimentar* **2018**, *32*, 26–30.
58. Castro, A.G.P. de Intervenção Educativa Para Promoção Do Consumo de Frutas E Hortaliças Sob a Perspectiva de Gestores de Unidades de Alimentação E Nutrição. Tese De Doutorado, Universidade de São Paulo - USP, 2011.
59. Cerrah, S.; Yigitoglu, V. Determining the Effective Factors on Restaurant Customers' Plate Waste. *International Journal of Gastronomy and Food Science* **2022**, *27*, 100469, doi:https://doi.org/10.1016/j.ijgfs.2022.100469.

60. Cook Hons, N.; Goodwin, D.; Collins, J.; Porter, J. "It's a Constant Changing Environment, and We're Just Playing Catch Up": Hospital Food Services, Food Waste, and COVID-19. *Nutrition & Dietetics* **2022**, *80*, doi:https://doi.org/10.1111/1747-0080.12762.
61. Corrêa, T.A.F.; Soares, F.B. da S.; Almeida, F.Q.A. de Índice de Resto-Ingestão Antes E Durante a Campanha Contra O Desperdício, Em Uma Unidade de Alimentação E Nutrição. *Higiene Alimentar* **2006**, *21*, 64–73.
62. Cristobal, J.; Castellani, V.; Manfredi, S.; Sala, S. Prioritizing and Optimizing Sustainable Measures for Food Waste Prevention and Management. *Waste Management* **2017**, *72*, 3–16, doi:https://doi.org/10.1016/j.wasman.2017.11.007.
63. Derqui, B.; Fernandez, V. The Opportunity of Tracking Food Waste in School Canteens: Guidelines for Self-Assessment. *Waste Management* **2017**, *69*, 431–444, doi:https://doi.org/10.1016/j.wasman.2017.07.030.
64. Di Pierro, R.; Frasnetti, E.; Bianchi, L.; Bisagni, M.; Capri, E.; Lamastra, L. Setting the Sustainable Development Targets for Restaurants and Italian HoReCa Sector. *Science of the Total Environment* **2023**, *855*, 158908, doi:https://doi.org/10.1016/j.scitotenv.2022.158908.
65. Diana, R.; Martianto, D.; Baliwati, Y.F.; Sukandar, D.; Hendriadi, A. Food Waste in Indonesian Hospitals: A Systematic Review. *Nutrition and Food Science* **2022**, *53*, 881–900, doi:https://doi.org/10.1108/NFS-05-2022-0150.
66. Dias-Ferreira, C.; Santos, T.; Oliveira, V. Hospital Food Waste and Environmental and Economic Indicators – a Portuguese Case Study. *Waste Management* **2015**, *46*, 146–154, doi:https://doi.org/10.1016/j.wasman.2015.09.025.
67. Djamaluddin, M.; Prawirohartono, E.P.; Paramastri, I. Analisis Zat Gizi Dan Biaya Sisa Makanan Pada Pasien Dengan Makanan Biasa. *Jurnal Gizi Klinik Indonesia* **2005**, *1*, 108, doi:https://doi.org/10.22146/ijcn.17379.
68. Dogdu, G.; Pekdemir, T.; Lakestani, S.; Karabörk, Ş.; Çavuş, O. Hidden Realities: Food Waste from Servings in Mini Size Packaging. *Waste Management* **2024**, *173*, 141–151, doi:https://doi.org/10.1016/j.wasman.2023.11.014.
69. Elnakib, S.A.; Quick, V.; Mendez, M.; Downs, S.; Wackowski, O.A.; Robson, M.G. Food Waste in Schools: A Pre-/Post-Test Study Design Examining the Impact of a Food Service Training Intervention to Reduce Food Waste. *International Journal of Environmental Research and Public Health* **2021**, *18*, 6389, doi:https://doi.org/10.3390/ijerph18126389.
70. Fassina, P.; Leonhardt , M.B.; Kerber, M. Análise Qualitativa Das Preparações Do Cardápio de Duas Unidades de Alimentação E Nutrição. *Arch. Health Sci. (Online)* **2019**, *26*, 153–157.
71. Fatkhurohman, F.; Lestari, Y.N.; Torina, D.T. Hubungan Perubahan Standar Porsi Makan Dengan Sisa Makanan Pasien Rumah Sakit Holistik Tahun 2016 (Studi Sisa Nasi Pada Menu Makan Siang Diet Di Rs Holistik). *Gizi Indonesia* **2017**, *40*, 1, doi:https://doi.org/10.36457/gizindo.v40i1.218.
72. Hannibal, B.; Vedlitz, A. Throwing It Out: Introducing a Nexus Perspective in Examining Citizen Perceptions of Organizational Food Waste in the U.S. *Environmental Science & Policy* **2018**, *88*, 63–71, doi:https://doi.org/10.1016/j.envsci.2018.06.012.
73. Heller, M.; Silva, A.B.G. da Manejo de Resíduos Em Uma Unidade de Alimentação E Nutrição. *Higiene Alimentar (Online)* **2015**, *29*, 191–193.

74. Heryawanti, P.T.; Prawirohartono, E.P.; Sudargo, T. Pengaruh Alat Penyajian Disposable Terhadap Sisa Makanan Pasien Di Ruang Rawat Inap RSUP Dr. Kariadi Semarang. *Jurnal Gizi Klinik Indonesia* **2004**, *1*, 78, doi:https://doi.org/10.22146/ijcn.17398.
75. Hidalgo Víquez, C.; Peña Vásquez, M. Cuantificación Del Desperdicio de Alimentos En Servicios de Alimentación de La Universidad de Costa Rica. *Perspectivas En Nutrición Humana* **2021**, *23*, 143–157, doi:https://doi.org/10.17533/udea.penh.v23n2a02.
76. Hopkins, L.C.; Webster, A.; Kennel, J.; Banna, J.; Bearden, D.; Finn, J.; Gunther, C. Plate Waste in USDA Summer Food Service Program Open Sites: Results from the Project SWEAT Sub-Study. *Journal of Hunger & Environmental Nutrition (Online)* **2023**, *18*, 699–712, doi:https://doi.org/10.1080/19320248.2023.2245783.
77. Iqbal, M.; Susetyowati, S.; Purba, M.B. The Effects of Room Service to Improve Patients' Food Satisfaction and Food Acceptance. *Gizi Indonesia* **2016**, *39*, 103, doi:https://doi.org/10.36457/gizindo.v39i2.213.
78. Irawati, I.; Prawiningdyah, Y.; Budiningsari, R.D. Analisis Sisa Makanan Dan Biaya Sisa Makanan Pasien Skizofrenia Rawat Inap Di Rumah Sakit Jiwa Madani Palu. *Jurnal Gizi Klinik Indonesia* **2010**, *6*, 123, doi:https://doi.org/10.22146/ijcn.17720.
79. Ismail, S.; Kadir, N.; Pusiran, A.K.; Zen, I.S.; Khan, A. The Importance of Menu Variety Experience for Public Health Sustainability at Higher Education Institution. *Indian Journal of Public Health Research & Development* **2019**, *10*, 1851, doi:https://doi.org/10.5958/0976-5506.2019.02724.4.
80. Jia, L.; Zhang, J.; Qiao, G. Scale and Environmental Impacts of Food Loss and Waste in China—a Material Flow Analysis. *International Journal of Environmental Research and Public Health* **2023**, *20*, 460, doi:https://doi.org/10.3390/ijerph20010460.
81. Jones, M.; Dailami, N.; Weitkamp, E.; Salmon, D.; Kimberlee, R.; Morley, A.; Orme, J. Food Sustainability Education as a Route to Healthier Eating: Evaluation of a Multi-Component School Programme in English Primary Schools. *Health Education Research* **2012**, *27*, 448–458, doi:https://doi.org/10.1093/her/cys016.
82. Khalid, S.; Malik, A.U.; Ullah, M.W.; Khalid, M.; Javeed, R.; Naeem, M.; Naseer, A. Food Waste: Causes and Economic Losses Estimation at Household Level in Pakistan. *Research Square* **2023** doi:https://doi.org/10.21203/rs.3.rs-1505062/v1.
83. Kodors, S.; Zvaigzne, A.; Litavniece, L.; Lonska, J.; Silicka, I.; Kotane, I.; Deksne, J. Plate Waste Forecasting Using the Monte Carlo Method for Effective Decision Making in Latvian Schools. *Nutrients* **2022**, *14*, 587, doi:https://doi.org/10.3390/nu14030587.
84. Lins, M.; Zandonadi, R.P.; Strasburg, V.J.; Nakano, E.Y.; Botelho, R.B.A.; Raposo, A.; Ginani, V.C. Eco-Inefficiency Formula: A Method to Verify the Cost of the Economic, Environmental, and Social Impact of Waste in Food Services. *Foods* **2021**, *10*, 1369, doi:https://doi.org/10.3390/foods10061369.
85. Longo-Silva, G.; Toloni, M.; Rodrigues, S.; Rocha, A.; Taddei, J.A. de A.C. Qualitative Evaluation of the Menu and Plate Waste in Public Day Care Centers in São Paulo City, Brazil. *Revista De Nutrição* **2013**, *26*, 135–144, doi:https://doi.org/10.1590/s1415-52732013000200002.
86. Love, D.C.; Asche, F.; Fry, J.P.; Nguyen, L.; Gephart, J.A.; Garlock, T.; Jenkins, L.D.; Anderson, J.L.; Brown, M.T.; Viglia, S.; et al. Aquatic Food Loss and Waste Rate in the United States Is Half of Earlier Estimates. *Nature Food* **2023**, *4*, 1058–1069, doi:https://doi.org/10.1038/s43016-023-00881-z.
87. Lu, M.-Y.; Ko, W.-H. Sustainable Preparation Behavior for Kitchen Staff in Order to Limit Food Waste. *Foods* **2023**, *12*, 3028–3028, doi:https://doi.org/10.3390/foods12163028.

88. Mardianingsih, N.; Utami, F.A.; Palupi, I.R. Capaian Standar Pelayanan Minimal Gizi Di Rumah Sakit Umum Daerah (RSUD) Manokwari Papua Barat. *Jurnal Gizi Klinik Indonesia* **2020**, *16*, 152, doi:https://doi.org/10.22146/ijcn.42425.
89. Martin-Rios, C.; Demen Meier, C.; Pasamar, S. Sustainable Waste Management Solutions for the Foodservice Industry: A Delphi Study. *Waste Management & Research: the Journal for a Sustainable Circular Economy* **2022**, *40*, 0734242X2210793, doi:https://doi.org/10.1177/0734242x221079306.
90. Martindale, W.; Schiebel, W. The Impact of Food Preservation on Food Waste. *British Food Journal* **2017**, *119*, 2510–2518, doi:https://doi.org/10.1108/bfj-02-2017-0114.
91. Mathisen, T.F.; Johansen, F.R. The Impact of Smartphone Apps Designed to Reduce Food Waste on Improving Healthy Eating, Financial Expenses and Personal Food Waste: Crossover Pilot Intervention Trial Studying Students' User Experiences. *JMIR Formative Research* **2022**, *6*, e38520, doi:https://doi.org/10.2196/38520.
92. Medeiros, M. das G.G. de A.; Castro, L.L.G. de; Bezerra, C. da S.; Penha, M.P. da; Lourenço, M.S. Avaliação Dos Serviços de Alimentação de Uma Universidade Pública Do Rio de Janeiro Na Pandemia de COVID-19. *Mundo saúde (Impr.)* **2022**, *46*, e12652022–e12652022.
93. Monteiro, M.A.M.; Cardoso, L.R.; Nogueira, M.; Schaeffer, M.A. Estudo Qualitativo E Quantitativo de Restaurantes Universitários Das Capitais Brasileiras. *Higiene Alimentar* **2014**, *28*, 73–78.
94. Musicus, A.A.; Vercammen, K.A.; Fulay, A.P.; Moran, A.J.; Burg, T.; Allen, L.; Maffeo, D.; Berger, A.; Rimm, E.B. Implementation of a Rooftop Farm Integrated with a Teaching Kitchen and Preventive Food Pantry in a Hospital Setting. *American Journal of Public Health* **2019**, *109*, 1119–1121, doi:https://doi.org/10.2105/ajph.2019.305116.
95. Ñunque G, M.; Salazar L, L.; Valenzuela A, C. Análisis Cuali-Cuantitativo de Menús Infantiles Ofrecidos En Restoranes Familiares Y de Comida Rápida En Santiago de Chile. *Revista Chilena De Nutrición* **2015**, *42*, 362–368, doi:https://doi.org/10.4067/s0717-75182015000400007.
96. O'Connor, T.; Kleemann, R.; Attard, J. Vulnerable Vegetables and Efficient Fishers: A Study of Primary Production Food Losses and Waste in Ireland. *Journal of Environmental Management* **2022**, *307*, 114498, doi:https://doi.org/10.1016/j.jenvman.2022.114498.
97. Ögel, İ.Y.; Ecer, F.; Özgöz, A.A. Identifying the Leading Retailer-Based Food Waste Causes in Different Perishable Fast-Moving Consumer Goods' Categories: Application of the F-LBWA Methodology. *Environmental Science and Pollution Research* **2023**, *30*, doi:https://doi.org/10.1007/s11356-022-24500-9.
98. Osei-Owusu, A.K.; Read, Q.D.; Thomsen, M. Potential Energy and Environmental Footprint Savings from Reducing Food Loss and Waste in Europe: A Scenario-Based Multiregional Input–Output Analysis. *Environmental Science & Technology* **2023**, *57*, 16296–16308, doi:https://doi.org/10.1021/acs.est.3c00158.
99. Palmer, S.; Herritt, C.; Cunningham-Sabo, L.; Stylianou, K.S.; Prescott, M.P. A Systems Examination of Food Packaging and Other Single-Use Item Waste in School Nutrition Programs. *Journal of Nutrition Education and Behavior* **2021**, *53*, 380–388, doi:https://doi.org/10.1016/j.jneb.2021.01.009.
100. Partearroyo, T.; Samaniego-Vaesken, M. de L.; Ruiz, E.; Aranceta-Bartrina, J.; Gil, Á.; González-Gross, M.; Ortega, R.M.; Serra-Majem, L.; Varela-Moreiras, G. Plate Waste Generated by Spanish Households and Out-of-Home Consumption: Results from the ANIBES Study. *Nutrients* **2020**, *12*, 1641, doi:https://doi.org/10.3390/nu12061641.
101. Pereira, I.G.S.; Lemos, L.W.; Lemos, K.G.E.; Akutsu, R. de C.C. de A.; Botelho, R.B.A.; Camargo, É.B. Construção E Implementação de Fichas Técnicas de Preparação de Unidade de Alimentação E Nutrição/

Construction and Implementation of Technical Datasheets for Preparation of Food and Nutrition Unit/ Construcción E Implementación de Fichas Técnicas... *Journal Health NPEPS* **2019**, *4*, 210–227.

102. Pettinger, C.; Smita, T.; Benji, S.; Gary, H. Collaborative Leadership to Support Sustainability in Practice for Dietitians as Allied Health Professionals. *Journal of Human Nutrition and Dietetics* **2023**, *36*, doi:https://doi.org/10.1111/jhn.13211.
103. Pinard, C.A.; Byker, C.; Serrano, E.; Harmon, A.H. National Chain Restaurant Practices Supporting Food Sustainability. *Journal of Hunger & Environmental Nutrition* **2014**, *9*, 535–545, doi:https://doi.org/10.1080/19320248.2014.898169.
104. Prescott, M.P.; Herritt, C.; Bunning, M.; Cunningham-Sabo, L. Resources, Barriers, and Tradeoffs: A Mixed Methods Analysis of School Pre-Consumer Food Waste. *Journal of the Academy of Nutrition and Dietetics* **2019**, *119*, 1270-1283.e2, doi:https://doi.org/10.1016/j.jand.2019.03.008.
105. Principato, L.; Ruini, L.; Guidi, M.; Secondi, L. Adopting the Circular Economy Approach on Food Loss and Waste: The Case of Italian Pasta Production. *Resources, Conservation and Recycling* **2018**, *144*, 82–89, doi:https://doi.org/10.1016/j.resconrec.2019.01.025.
106. Razalli, N.H.; Cheah, C.F.; Mohammad, N.M.A.; Abdul Manaf, Z. Plate Waste Study among Hospitalised Patients Receiving Texture-Modified Diet. *Nutrition Research and Practice* **2021**, *15*, 655, doi:https://doi.org/10.4162/nrp.2021.15.5.655.
107. Rochmah, T.N. Improving Nutrition Services to Reduce Plate Waste in Patients Hospitalized Based on Theory of Constraint. *Amerta Nutrition* **2020**, *4*, 335, doi:https://doi.org/10.20473/amnt.v4i4.2020.335-341.
108. Roe, B.E.; Apolzan, J.W.; Qi, D.; Allen, H.R.; Martin, C.K. Plate Waste of Adults in the United States Measured in Free-Living Conditions. *PLOS ONE* **2018**, *13*, e0191813, doi:https://doi.org/10.1371/journal.pone.0191813.
109. Roe, B.E.; Qi, D.; Apolzan, J.W.; Martin, C.K. Selection, Intake, and Plate Waste Patterns of Leftover Food Items among U.S. Consumers: A Pilot Study. *PLOS ONE* **2020**, *15*, e0238050, doi:https://doi.org/10.1371/journal.pone.0238050.
110. Schwartz, M.B.; Henderson, K.E.; Read, M.; Danna, N.; Ickovics, J.R. New School Meal Regulations Increase Fruit Consumption and Do Not Increase Total Plate Waste. *Childhood Obesity* **2015**, *11*, 242–247, doi:https://doi.org/10.1089/chi.2015.0019.
111. Silva, E.S.; Januário, B.L.; Silveira, M.A.R.; Scaglioni, F.T.; Silva, Z.M. da Avaliação Do Desperdício de Hortifrutis Em Unidade de Alimentação E Nutrição Na Cidade de São Paulo, SP. *Higiene Alimentar* **2013**, *27*, 24–30.
112. Strasburg, V.J.; Prattes, G.; Acevedo, B.; Suárez, C. Calidad Nutricional E Impacto En Medio Ambiente Por Los Insumos de Un Comedor Universitario En Uruguay. *Archivos Latinoamericanos De Nutricion* **2023**, *73*, 90–101, doi:https://doi.org/10.37527/2023.73.2.001.
113. Strotmann, C.; Baur, V.; Börnert, N.; Gerwin, P. Generation and Prevention of Food Waste in the German Food Service Sector in the COVID-19 Pandemic – Digital Approaches to Encounter the Pandemic Related Crisis. *Socio-Economic Planning Sciences* **2022**, *82*, 101104, doi:https://doi.org/10.1016/j.seps.2021.101104.
114. Syauqiyatullah, A., Anwar, I.Z., Darmarini, F., & Syauqy, A. Analisis faktor-faktor penyebab sisa makanan cair pasien kelas 2 dan 3 di gedung a rsupn dr. cipto mangunkusumo jakarta tahun 2019. 2020, doi: 10.36457/GIZINDO.V43I2.472.

115. Tanuwijaya, L.K.; Sembiring, L.G.; Dini, C.Y.; Arfiani, E.P.; Wani, Y.A. Sisa Makanan Pasien Rawat Inap: Analisis Kualitatif. *Indonesian Journal of Human Nutrition* **2018**, *5*, 51–61, doi:https://doi.org/10.21776/ub.ijhn.2018.005.01.6.
116. Tenser, C.M.R.; Ginani, V.C.; Coelho, A. Ações Contra O Desperdício Em Restaurantes E Similares. *Higiene Alimentar* **2007**, *21*, 22–25.
117. Whitehair, K.J.; Shanklin, C.W.; Brannon, L.A. Written Messages Improve Edible Food Waste Behaviors in a University Dining Facility. *Journal of the Academy of Nutrition and Dietetics* **2013**, *113*, 63–69, doi:https://doi.org/10.1016/j.jand.2012.09.015.
118. Wilkie, A.; Graunke, R.; Cornejo, C. Food Waste Auditing at Three Florida Schools. *Sustainability* **2015**, *7*, 1370–1387, doi:https://doi.org/10.3390/su7021370.
119. Yazdankhah, Z.; Mehrabi, Y.; Rakhshanderou, S.; Safari-Moradabadi, A.; Ghaffari, M. Behavioral Approach to Food Consumption and Waste Production: A Quasi-Experimental Study. *Journal of Education and Health Promotion* **2020**, *9*, 343, doi:https://doi.org/10.4103/jehp.jehp_506_20.
